# Metatranscriptomic Analysis Reveals SARS-CoV-2 Mutations in Wastewater of the Frankfurt Metropolitan Area in Southern Germany

**DOI:** 10.1128/MRA.00280-21

**Published:** 2021-04-15

**Authors:** Shelesh Agrawal, Laura Orschler, Susanne Lackner

**Affiliations:** a Technical University of Darmstadt, Institute IWAR, Chair of Wastewater Engineering, Darmstadt, Germany; DOE Joint Genome Institute

## Abstract

We report a sequencing analysis of severe acute respiratory syndrome coronavirus 2 (SARS-CoV-2) RNA in wastewater samples collected in the Frankfurt metropolitan area of Germany. The majority of the detected mutations have been identified only in clinical genomes outside Frankfurt, indicating that the sequencing of SARS-CoV-2 RNA in wastewater can provide insights into emerging variants in a city.

## Abstract (precis)

We report a sequencing analysis of severe acute respiratory syndrome coronavirus 2 (SARS-CoV-2) RNA in wastewater samples collected in the Frankfurt, Germany, metropolitan area. The majority of the detected mutations have been identified only in clinical genomes outside Frankfurt, indicating that the sequencing of SARS-CoV-2 RNA in wastewater can provide insights into emerging variants in a city.

## ANNOUNCEMENT

Wastewater-based epidemiology (WBE) can provide complementary information about the dynamics of severe acute respiratory syndrome coronavirus 2 (SARS-CoV-2) infections at the community level because SARS-CoV-2 (genus *Betacoronavirus*, family *Coronaviridae*) RNA has been detected in human urine and feces (1, 2). However, most of the WBE studies have focused primarily on reverse transcription-quantitative PCR analysis (3–5). Since the onset of the coronavirus disease 2019 (COVID-19) pandemic, several clinically relevant mutations have been reported, resulting in various SARS-CoV-2 variants (6–9). A few studies showed that the sequencing of SARS-CoV-2 RNA in wastewater could help to determine which SARS-CoV-2 variants are circulating in the studied areas (10, 11).

We collected 24-h composite samples on 4 December 2020 at three sampling points in the Frankfurt metropolitan area of Germany. The three sampling points were (i) influent of the wastewater treatment plant (WWTP) Niederrad (NR) (50.08N, 8.63E), (ii) influent of the WWTP Sindlingen (SL) (50.09N, 8.51E), and (iii) a sewage sample from Griesheim (GR) (50.09N, 8.60E). One liter of the untreated wastewater was filtered through a 0.45-μm electronegative membrane filter to concentrate the virus-like particles, followed by extraction using the FastRNA Pro Blue kit (MP Biomedicals) according to the manufacturer’s protocol. cDNA synthesis was performed using SuperScript VILO Master Mix (Thermo Fisher Scientific), followed by library preparation using the Ion AmpliSeq SARS-CoV-2 research panel (Thermo Fisher Scientific) according to the manufacturer’s instructions. This panel consists of 237 primer pairs, resulting in an amplicon length range of 125 to 275 bp and covering the nearly full genome of SARS-CoV-2. Libraries were multiplexed and sequenced using an Ion Torrent 530 chip on an Ion S5 sequencer (Thermo Fisher Scientific) according to the manufacturer’s instructions.

Ion Torrent Suite software (v 5.12.2) of the Ion S5 sequencer was used to map the generated reads to a SARS-CoV-2 reference genome (Wuhan-Hu-1 [GenBank accession numbers NC_045512 and MN908947.3]), using TMAP software included in the Torrent Suite. The sequencing data for the samples are summarized in Table 1. For mutation calls, additional Ion Torrent plugins were used. First, all single-nucleotide variants (SNVs) were called using Variant Caller (v5.12.0.4) with “Generic - S5/S5XL (510/520/530) - Somatic - Low Stringency” default parameters. Then, for annotation and determination of the base substitution effect, COVID19AnnotateSnpEff (v1.0.0.1), a plugin developed explicitly for SARS-CoV-2, was used. To determine whether the mutations detected in these samples had already been reported in clinical samples at different locations, we downloaded the clinically reported mutations and their corresponding locations from COVID CG (https://covidcg.org) (12) on 7 March 2021.

**TABLE 1:** Summary of sequencing data for the samples

Sample	Sample location	Total no. of reads	No. of mapped reads	Avg target base coverage depth (×)	Avg read identity to target (%)^*a*^	GC content (%)	Accession no.
NR	Niederrad	2,781,914	854,804	817.9	95.68	48.3	SAMN18310569
SL	Sindlingen	2,143,766	675,006	680.8	94.01	46.3	SAMN18310571
GR	Griesheim	2,349,476	640,702	215.1	94.84	47.4	SAMN18310570

a The target sequence was the SARS-CoV-2 reference genome (Wuhan-Hu-1 [GenBank accession numbers NC_045512 and MN908947.3]).

**FIG 1:**
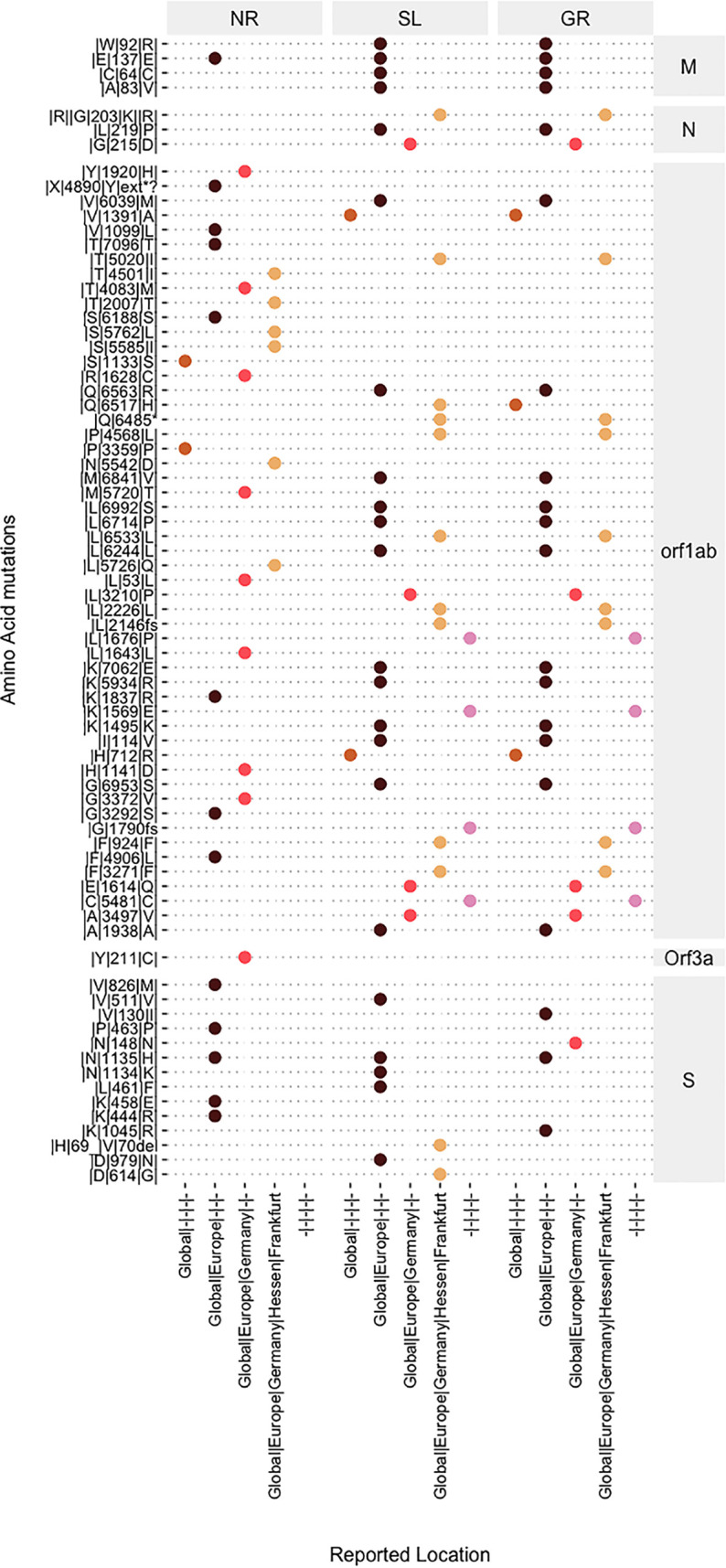
Locations of mutations detected in samples (NR, Niederrad; SL, Sindlingen; GR, Griesheim), corresponding to the locations reporting the mutations in clinical samples. The detected mutations are organized according to the corresponding gene (M, N, open reading frame 1ab [Orf1ab], Orf3a, and S) of the SARS-CoV-2 genome. Points indicate the locations reporting the detected mutations.

We detected 75 unique mutations across all of the samples, including 5 mutations reported outside Europe, 35 reported in Europe, 13 reported in Germany, 18 reported in the Frankfurt region, and 4 not reported (Fig. 1). In the SL sample, we also observed D614G, a predominant mutation reported around the globe (13) and a key mutation associated with the B.1.1.7, B.1.351, and P.1 variants (14). The 69/70 deletion mutation, a key mutation associated with B.1.1.7 (14), was also found in the SL sample. Due to the plausible presence of SARS-CoV-2 RNA of multiple variants in wastewater, WBE analysis can provide only indirect information about the variants. However, observations from this study suggest that an integration of genomic epidemiology into WBE may support identification of variants that have already been detected in a city, as well as those that have not yet been detected in clinical samples.

### Data availability.

The raw metagenomic sequence data are available in the NCBI Sequence Read Archive (SRA) under BioProject number PRJNA714504 and SRA accession numbers SRX10340342 (NR), SRX10340344 (SL), and SRX10340343 (GR).
